# Screening for genes that wire the cerebral cortex

**DOI:** 10.1186/1741-7007-9-1

**Published:** 2011-01-07

**Authors:** Ludmilla Lokmane, Sonia Garel

**Affiliations:** 1 Ecole Normale Supérieure, Institut de Biologie de l'ENS, IBENS, 46 rue d'Ulm, 75230 Paris cedex 05, France; 2 INSERM, U1024, Avenir Team, 46 rue d'Ulm, 75230 Paris cedex 05, France; 3 CNRS, UMR 8197, 46 rue d'Ulm, 75230 Paris cedex 05, France

## Abstract

Thalamocortical projections convey visual, somatosensory and auditory information to the cerebral cortex. A recent report in *Neural Development *shows how a forward genetic screen has enabled the identification of novel mutations affecting specific decision points of thalamocortical axon pathfinding.

See research article: http://www.neuraldevelopment.com/content/6/1/3/abstract

## Commentary

Understanding how the brain becomes wired-up during development is essential not only to gain insight into its normal functioning, but also to progress in the comprehension of neurological and psychiatric disease. Indeed, there is increasing evidence that defects occurring during embryonic development lead to impaired functioning of the cerebral cortex, and that such defects may underlie the etiology of several human pathologies, including schizophrenia and autism spectrum disorders. Genetic studies in both humans and mice have enabled clinicians and neurobiologists to dissect molecular cascades that govern the formation of the cerebral cortex, in particular those directing the generation and migration of different populations of cortical neurons. However, similar large-scale unbiased studies have yet to be developed to study the formation of the major axonal tracts that wire the cerebral cortex.

## The dorsal thalamus: a sensory gateway of the brain

**Figure 1:**
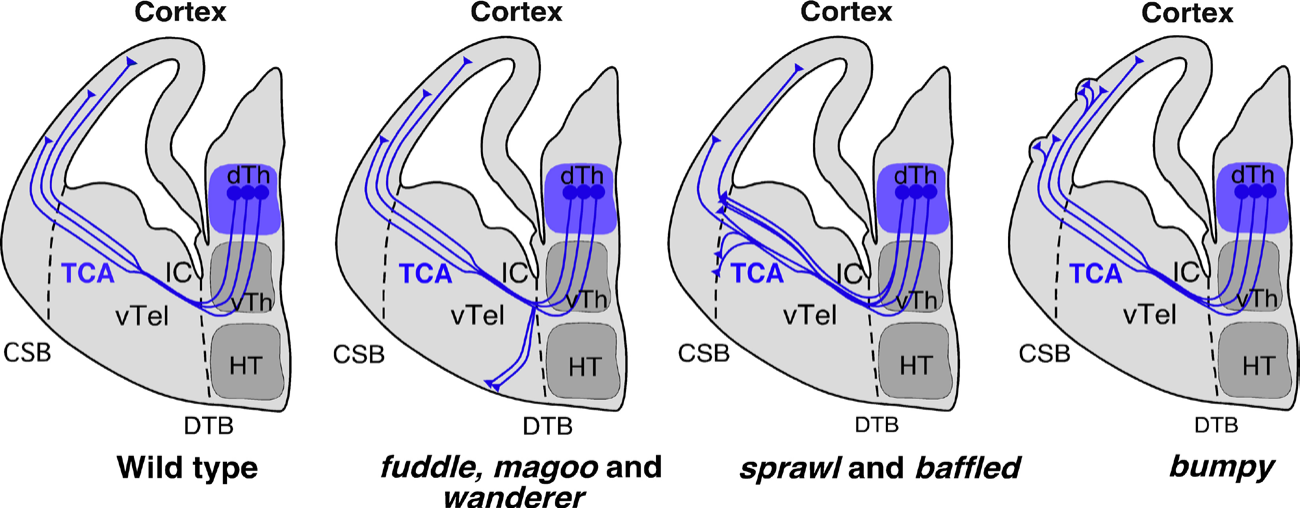
**The paths of thalamocortical axons in wild-type and mutant embryos**. Schematic representations of coronal sections of one side of the mouse brain at embryonic day 16.5 (E16.5). In wild-type embryos, the thalamocortical axons (TCA) extend ventrally from the dorsal thalamus (dTh), turn and cross the diencephalon-telencephalon boundary (DTB), enter the ventral telencephalon (vTel) and grow into the internal capsule (IC) where they separate into distinct fascicules. They then cross the cortico-striatal boundary (CSB) and turn dorsally into the cortex. In *fuddle*, *magoo *and *wanderer *mutants the axons are partially misrouted ventrally at the DTB. In *sprawl *and *baffled *mutants the axons are overfasciculated and are stalled at the CSB. In the *bumpy *mutant some axons aberrantly invade the marginal zone to innervate ectopic clusters of cells. HT, hypothalamus; vTh, ventral thalamus.

The dorsal thalamus is a sensory gateway of the brain that receives visual, somatosensory and auditory information. Thalamocortical axons convey this sensory information from the dorsal thalamus to the cerebral cortex and hence are essential to brain function. They follow a long and complex path to reach their final cortical targets, making successive changes in direction as they navigate from one intermediate target to another (Figure 1). Indeed, from embryonic day 13 (E13) in mice, dorsal thalamus axons extend ventrally, turn laterally close to the hypothalamus to cross the boundary between embryonic diencephalon and telencephalon, enter the ventral telencephalon, grow in the internal capsule, and fan out into smaller axonal bundles before crossing the cortico-striatal boundary at E15. The axons then turn dorsally into the intermediate zone of the cerebral cortex, where they interact with cells of the cortical subplate before extending collateral branches to reach their final target in layer IV of the cerebral cortex [1].

The cellular and molecular mechanisms that control this long axonal journey have been a puzzle for many years. Analyses of several mutant mouse lines have revealed that transcription factors, guidance cues, adhesion molecules and guidepost cells control the navigation of thalamocortical axons at specific decision points within their intermediate targets [1-5]. For instance, expression of the neuronal guidance protein Slit2 in the hypothalamus is required to prevent the thalamocortical axons from entering this structure and promotes their turning into the ventral telencephalon [2,3]. In parallel, guidepost cells in the ventral thalamus and telencephalon that extend transient projections have also been proposed to guide the axons along their journey [4,6]. In the ventral telencephalon, tangential neuronal migration allows the formation of a permissive corridor required for the thalamocortical pathfinding within this structure [7]. However, the precise molecular and cellular mechanisms controlling the successive steps of thalamocortical axon pathfinding are still not fully understood.

## A forward genetic screen to identify new genes controlling thalamocortical pathfinding

In a paper published recently in *Neural Development*, Dwyer *et al*. [8] report the results of an unbiased forward genetic screen for thalamocortical axon pathfinding mutants using a transgenic reporter line of mice that they generated - ThalamoCortical Axon-*tau-LacZ *(*TCA-TLZ*). In these mice, the reporter protein beta-galactosidase is expressed strongly and reliably from E13.5 onward in dorsal thalamus neuronal cell bodies as well as in their axons [8]. The *TCA-TLZ *line therefore allows the visualization of the entire population of thalamocortical axons, thereby providing an elegant genetic tool to examine the global navigation and connectivity of the thalamocortical system. Dwyer *et al*. subjected mice of the *TCA-TLZ *line to random mutagenesis with N-ethyl-N-nitrosourea (ENU), followed by a breeding strategy of two intercrosses and one backcross, in order to identify thalamocortical axon phenotypes in homozygote mutant embryos at E18.5. Indeed, this experimental approach had the unique advantage of making it possible to screen for the effects of recessive mutations, including potentially lethal ones, on the whole brain [9].

Amongst 57 lines screened for an abnormal pattern of *TCA-TLZ *expression, the authors isolated 6 independent recessive mutant lines whose mutations were mapped using an experimental procedure previously developed to identify mouse models of human pathologies. Interestingly, the high proportion of mutant lines identified (6 out of 57) indicates that thalamocortical axon navigation is a very sensitive process, most probably due to the fact that these axons cross several intermediate targets. Therefore, the analysis of defects in this system not only provides essential knowledge about novel genes controlling the formation of these particular projections, but also constitutes a selective read-out of abnormal forebrain morphogenesis.

Remarkably, out of the six mutant lines identified, five carried mutations that mapped to chromosomal regions not previously known to contain genes involved in thalamocortical axon development. Dwyer *et al*. found that these six mutations lead to defects in several critical decision points (Figure 1). In *fuddle *and *magoo *mutants, the axons were partially misrouted ventrally in the ventral telencephalon just after crossing the diencephalon-telencephalon border. In *sprawl *and *baffled *mutants, the axons were overfasciculated and disorganized in the internal capsule, and they also stalled at the cortico-striatal boundary. In *bumpy *mutants, thalamocortical axons abnormally innervated ectopic clusters of cells in the marginal zone of the cerebral cortex. In addition, Dwyer *et al*. found a sixth mutant, *wanderer*, with thalamocortical pathfinding defects, in which a subset of axons was misrouted ventrally in the ventral telencephalon (Figure 1). The genetic mapping of *wanderer *revealed that the phenotype is caused by a novel mutation in the *Emx2 *gene, which encodes a transcription factor already known to control cortical development and thalamocortical axon guidance in mice [10]. Using the *TCA-TLZ *reporter line, the authors observed that in this *Emx2 *mutant the ventrally misrouted axons grew rostrally along the lateral olfactory tract, a behavior that had not been visualized before [10]. Indeed, thalamocortical axon pathfinding has so far been mostly examined by either axonal tracing using lipophilic tracers or immunostaining, neither of which enable a specific labeling of the entire axonal projection. This novel analysis of *wanderer*/*Emx2 *mutants highlights the fact that the robustness of the *TCA-TLZ *reporter allows the visualization of phenotypes that could not be detected otherwise, and that ventrally misrouted thalamocortical axons can aberrantly join another large axonal tract when they encounter it. Moreover, heterozygous mutations of *EMX2 *in humans have been implicated in some cases of a rare developmental disorder, schizencephaly, characterized by different clinical features, including mental retardation, hypotonia, epilepsy and spasticity [11,12]. Thus, the identification of another mutation in the *Emx2 *gene validates the forward genetic screen as an efficient way of identifying genes controlling forebrain development in the mouse and those with a potential involvement in human pathologies.

Identifying the genes mutated in *magoo*, *fuddle*, *sprawl*, *baffled *and *bumpy *and understanding their function is the next challenge. In the meantime, the work of Dwyer *et al*. highlights the feasibility and major importance of forward genetics in mice to identify novel genes involved in thalamocortical axon and forebrain development and opens the way for future screens to unravel the molecular mechanisms controlling brain wiring.
